# Impact of Sudden Cardiac Arrest and Implantable Cardiac Defibrillator Shocks on Mental Health: Review and Management Guidance

**DOI:** 10.1016/j.jacadv.2025.101797

**Published:** 2025-05-14

**Authors:** Estelle Torbey, Carlos Mena-Hurtado, Jamie L. Jackson, Zainab Samaan, Samuel F. Sears, Susanne S. Pedersen, Anurada Lala, Rosy Thachil, Jill Steiner, Onyedika J. Ilonze, Davis Jones, Karol Watson, Andrea Price, Christopher Knoepke, Jim W. Cheung, Kim Smolderen, ACC Cardiopsychology Work Group

**Affiliations:** a Department of Internal Medicine, Warren Alpert Medical School, Brown University, Providence, Rhode Island, USA; b Department of Internal Medicine, Yale University, New Haven, Connecticut, USA; c Center for Biobehavioral Health, Nationwide Children's Hospital, Columbus, Ohio, USA; d Department of Psychiatry, Queen's University, Kingston, Ontario, USA; e Department of Psychology, East Carolina University, Greenville, North Carolina, USA; f Departments of Psychology and Cardiovascular Sciences, University of Southern Denmark, Odense, Denmark; g Department of Cardiology, Odense University Hospital, Odense, Denmark; h Department of Internal Medicine, Mount Sinai Icahn School of Medicine, New York, New York, USA; i Department of Internal Medicine, University of Washington Medical Center, Seattle Washington, USA; j Division of Cardiovascular Medicine, Krannert Cardiovascular Research Center, Indiana University, Indianapolis, Indiana, USA; k Department of Cardiology, UCLA School of Medicine, Los Angeles, California, USA; l Indiana University Health System, Indianapolis, Indiana, USA; m Department of Psychology, University of Colorado, Denver, Colorado, USA; n Department of Internal Medicine, Weill Cornell, New York, New York, USA

**Keywords:** ICD shock, mental health, SCA

## Abstract

Survivors of sudden cardiac arrest (SCA) and those who experience shocks from an implantable cardiac defibrillator (ICD-S) are at risk of developing unrecognized and untreated mental health (MH) symptoms. MH sequelae can include anxiety, depression, or post-traumatic stress symptoms which hinder one's ability to return to usual life and activity, impeding follow-up, health care seeking, and adherence to care plans. Addressing MH as part of a whole person care in such scenarios could lead to improved wellness and recovery. This review examines the MH sequelae of SCA and ICD-S, explores potential therapies for managing these issues, proposes strategies to improve MH post-SCA or defibrillator shock, and identifies areas for future research.

## Highlights

• MH disorders post-SCA and ICD-S are underdiagnosed and there is a lack of standards for integrated care pathways that attend to whole person functioning.
• Management and diagnosis require patient and family care and education, multidisciplinary collaborative efforts, with alternative payment models, and linkage to community partners.
• Gaps in research and integrating existing therapies are necessary to overcome barriers to effective management pathways.
• Standardized pathways that are system wide are needed for behavioral, cognitive, and complementary therapy for patients and their families, ensuring that both patients and their families receive adequate support after SCA and ICD-S.

Sudden cardiac arrest (SCA) and the experience of implantable cardiac defibrillator (ICD) shocks (ICD-S) can have a profound psychological impact on the patients' mental health (MH) and recovery. Anxiety, depression, and post-traumatic stress symptoms can occur and impact their quality of life (QoL),^1^ including those with relatively good neurological recovery able to express their fear and anxiety.^2^ While survival after SCA has increased over the years, there are limited guidelines on postresuscitation mental health care (MHC).^3^ The potential for gaps in care can leave patients without viable recovery plans, requiring them to use their own resources for support during recovery. Increasingly, integrated MHC in cardiovascular service lines is called for but is only available in limited settings.^4^^,^^5^ Integrated MHC involves the provision of efficient care that responds to the whole of a patient's health needs (including physical, psychosocial, and MH working jointly with the patient, and family members.^6^ Most patients and their families, post-SCA or ICD-S, are likely to benefit from a range of direct and indirect integrated care options to promote the psychological and spiritual well-being for patients and their families and to achieve better health states.^7^ Integrating MHC pathways into the post-SCA treatment pathway helps create plans for the whole-person's well-being and is consistent with a population health approach.^8^ We present in this narrative review article the current knowledge surrounding MHC post-ICD-S and SCA as well as the gaps and solutions to developing a comprehensive treatment pathway (Central Illustration).

**Central Illustration:**
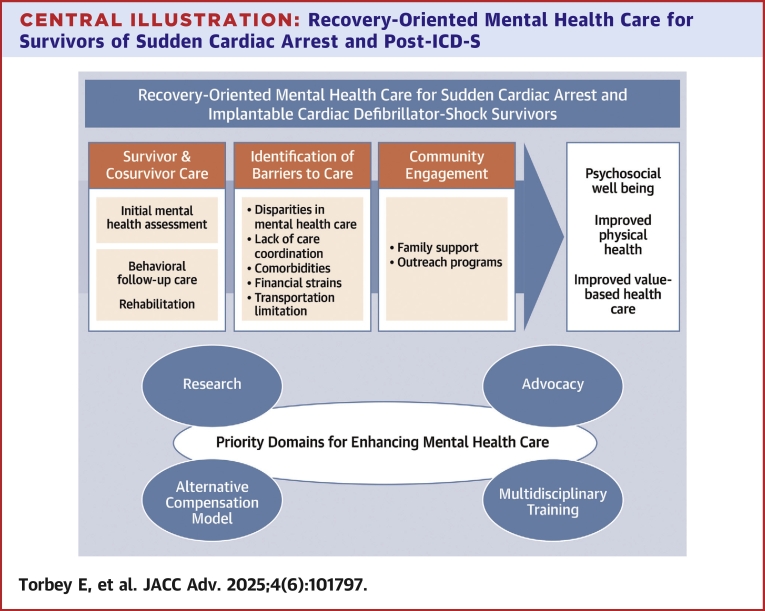
Recovery-Oriented Mental Health Care for Survivors of Sudden Cardiac Arrest and Post-ICD-S Figure illustrating steps toward recovery-oriented mental health care for SCA and ICD shock survivors, enhancing physical health and value-based care by addressing mental health gaps. Abbreviations as in Figure 2.

## Effect of ICD-S on MH

**Figure 1:**
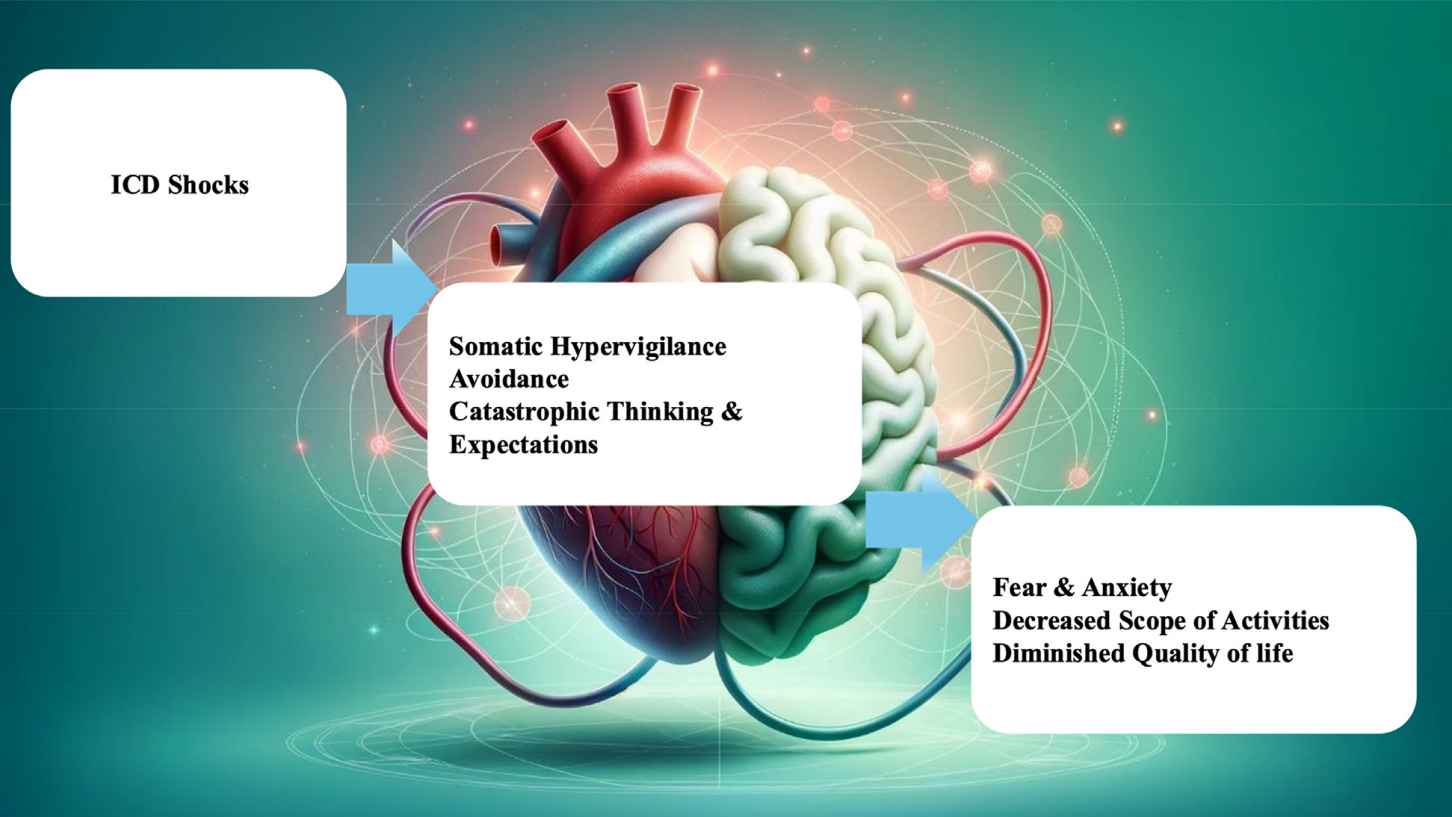
Anxiety Components Post-ICD-S Diagram depicting the main behavioral symptoms in the setting of anxiety post-ICD-S.

ICDs reduce mortality among appropriately selected patients who are at high risk for SCA. ICD-S can represent a unique threat and a pain stimulus to which patients respond with a specific anxiety response (Figure 1). In a large meta-analysis by Ghezzi et al, patients with ICDs developed disorders of anxiety, depression, and post-traumatic stress disorder (PTSD) in 23%, 15%, and 12%, respectively.^1^ ICD-S can significantly impact a patient's psychological well-being by adding to the stress of having a cardiac disease^9^ and an ICD, particularly among patients with preexisting MH disorders.^10^ Furthermore, anxiety has been established as a unique stressor, beyond general distress, for patients with ICD-S^7^^,^^11^ occurring in 15% to 44% of patients,^11^ and lasting up to 2 years after the initial shock.^12^ Phantom shocks, defined as similar bodily sensations to a shock without any device activation, can occur in up to 5% post-ICD-S and can further exacerbate these symptoms.^13^^,^^14^

Even in asymptomatic patients, the possibility or anticipation of a shock may be enough to create significant shock anxiety. In a follow-up study of the MADIT-RIT (Multicenter Automatic Defibrillator implantation Trial-Reduce Inappropriate Therapy), ≥2 appropriate or inappropriate ICD-S or appropriate antitachycardia pacing therapies were associated with shock-related anxiety at 9-month follow-up.^15^ While both appropriate and inappropriate ICD-S can lead to stress, higher numbers of inappropriate shocks are associated with higher anxiety score.^15^ An inappropriate shock is perceived as a traumatic error rather than a life-saving intervention. This perceived lack of necessity, unpredictability, and potential for distrust in the device that leads to a heightened constant fear and hypervigilance,^16^ emphasizing the importance of appropriate device programming.^17^

Lack of information exacerbates fear and insecurity in both patients and their families post-ICD-S.^10^ Setting explicit expectations regarding ICD therapies, making shared decision, and using the available clinical decision aid tools such as the Colorado shared decision tool^18^ preimplant and during subsequent visits can decrease these fears. Interventions to involve and empower family members and caregivers remain a critical need in this area. Studies comparing the inclusion of patients' partners to treatment plans vs patients alone showed further reduction in anxiety.^19^^,^^20^

Additional psychological support such as cognitive behavioral therapy (CBT), either for anxiety or for depression, and other trauma-focused therapies may also be critical to mitigate adverse effects and enhance the patient's overall well-being, especially as it relates to the traumatic shocks one may have experienced or is anticipating.^19^, ^20^, ^21^ Therefore, regular assessment and follow-up have maintained integral importance in these patients' MH. Importantly, the nature of anxiety and depression symptoms, their intensity and their impact on various life domains require further clinical assessment. Indications of treatments and treatment preferences will need to be discussed with the behavioral health clinician and the patient. Management of patients with ICDs, however, has shifted to remote monitoring over in person clinic visits, leaving patients unable to discuss fears and anxieties with providers. Studies have examined the use of web-based psychological support interventions to manage anxiety or depression post-ICD-S, however results have been mixed in terms of efficacy.^22^^,^^23^

These fears extend to the development of avoidance behaviors (including exercise avoidance) that can impact social integration and activity levels due to the anxiety of tachycardia and triggering and ICD-S.^24^ Cardiac rehabilitation (CR) programs^25^ and somatic practices such as yoga,^26^ when available and affordable, could potentially safely and adjunctively address these fears. Specifically, CR has shown to increase self-reported general health scores, a combination of physical and emotional well-being.^27^ However, access to CR is not offered to every patient after an ICD-S and many patients are limited in their ability to participate due to work or other social needs.^28^ Providing CR referrals in the in-patient setting can increase participation rates and overall improved MH.^28^

In summary, a multidisciplinary team can achieve comprehensive supportive care with initial ICD patients' education and engagement and subsequent adapted MHC using variable methods of delivery (telehealth, group-based, individual therapy). Care should be focused on early enactment of these services, regular follow-up, as well as involvement of family members.

## Effect of SCA on MH

The burden of SCA is significant on the U.S. health system and is the leading cause of death in Western countries with an incidence of 15% to 20% of all deaths with a particular uptick in numbers due to the COVID-19 pandemic.^29^^,^^30^ Surviving SCA has important implications for the MH and QoL of patients and their families which can substantially impact future cardiac and general care. Initiation of post-SCA medications with behavioral side effects, such as antiepileptics, physical impairment, and restriction of activity resulting from brain injury, development of significant comorbidities such as renal and heart failure, and socioeconomic barriers to accessing psychological support are all factors that affect the MH of survivors of SCA (SSCA).^31^ SSCA tend to have daily somatic remainders of their near-death experience with increases in heart rate or chest pains, all worsening MH.^32^ These symptoms partially mirror those seen in postcritical illness syndrome, with the initial pooled prevalence of depression at 19%, rising to 30% after 1 year.^32^ One study showed a significant increase in mortality over 14 years in SSCA with depression or anxiety compared to SSCA without depression or anxiety (36% vs 27%; *P* = 0.001).^33^ Therefore, screening for depression and anxiety is important as it is essential to provide adequate care and establish individualized treatment plans. Symptoms interfere with QoL and the ability to follow-up with medical care. They can even predispose to suicidal ideation in up to 15% of patients surviving in-hospital SCA.^34^ PTSD is more common after SCA than after stroke or acute coronary syndromes due to its sudden nature and often prolonged intensive care unit stays.^35^ Furthermore, PTSD, and more specifically the hyperarousal component, triples the risk of recurrent major adverse cardiovascular events and all-cause mortality.^36^ Interoceptive hypersensitivity and exaggerated interpretations leading to hyperarousal have been associated with increased sympathetic tone as well as cardiovascular reactivity, and degraded sleep quality.^37^

The collateral effect of the SCA event on family members needs to be taken into consideration while developing short- and long-term management plans for MHC as they are cosurvivors of these traumatic events. MH outcomes for family members relate to variability in trauma exposure^38^ (eg, presence during resuscitation, prognostic uncertainty), adjustment difficulties due to perceived changes in the SSCA's abilities (eg, increased dependence, role disturbances, neurocognitive sequelae), experiences with accessing services (eg, communication by medical team, postdischarge care) as well as the implications of genetic screening on the family members.^39^ Collectively, the needs of family members are identifiable, but viable and sustainable tactics to address them have proven challenging without cohesive networks of care. Studies have shown that families of SSCA benefit from improved communication between provider's and family, early debriefing and support interventions, as well as long-term MHC.^40^ Furthermore, both SSCA and families find a steep-learning curve with life post event (ie, expectations in recovery, ability to work) to which early (within 1 week) support after discharge is imperative.^41^

The effect of SCA on MH is not all negative as post-traumatic growth has been described as well. A prospective study of 110 SSCA^42^ examined emotional distress and positive psychological factors nearing hospital discharge. Mindfulness (ie, living in the present moment) and perceived social support were associated with less depressive, anxiety, and PTSD symptoms. Furthermore, existential well-being (ie, how meaningful one perceives their life) and resilient coping (ie, adaptability) were associated with less depressive and PTSD symptoms. While more research is needed, these findings suggest that interventions to enhance positive psychological experience may be considered to improve post-SCA well-being.^42^

## Psychological interventions post-SCA

### Nonpharmacological therapy

Psychological care post-SCA is uncommon worldwide and lacks clinical practice guidelines for management.^32^ CBT remains the mainstay therapy: in its first and second wave, it allows identification of ensuing negative thoughts and emotions and encourages patients to engage in meaningful activity as well as relaxation training. A retrospective case review of young SCA patients (n = 251) in France revealed that only 12% were referred for MHC^43^ with 83.2% of SSCAs and families stating a psychologist should be included in the follow-up care.^44^ CR can be an optimal setting where CBT could be integrated. The multidisciplinary approach used in CR includes specific attention to the MH, making this an appealing setting for post-SCA or ICD-S intervention. However, there is a lack of evidence for its use and further dedicated research is needed to support the use of CR in the nonmyocardial infarction SCA.

Mind and body interventions are known to have roles in attenuating autonomic stress, and their benefit in SSCAs remains to be determined. A qualitative study of SSCAs highlighted an ongoing effort to “make sense” of SCA, trust mind and body recovery, and life reevaluation that can result in a “new normal.”^41^ A novel strategy that combines exposure therapy and mindfulness was found to improve PTSD in 80% of SSCAs.^45^ Another parasympathetic-enhancing technique that is being assessed to decrease PTSD in SSCA is slow diaphragmatic breathing at 0.1 Hz driven by an application with live feedback and heart monitoring.^46^^,^^47^ Inhibition of the sympathetic system by stellate ganglionic block is another intervention currently being assessed in SSCA after its proven benefit in veterans with PTSD.^48^

Additionally, complementary interventions such as yoga and support groups can improve anxiety symptoms. Though some evidence suggests that yoga is beneficial in the treatment of cardiac arrhythmia,^49^ studies have not shown its superiority to CBT and the role of yoga-based rehabilitation in managing mental distress post-SCA is yet to be determined.^50^

Group therapy provides supportive environments for individuals to share experiences, learn coping strategies, and build social connections that lead to improved MH. In general, these formats also reduce feelings of isolation while increasing a sense of belonging and enhancing coping skills.^14^ Peer support groups for SSCA have been proven to be beneficial, especially in younger patients, allowing a place of safety to discuss shared events, creating some sort of “sense-making” of the event, as well as providing hope into the future.^51^^,^^52^

Focus on spirituality as another complementary strategy includes considerations for beliefs and values, meaning and connections, self-transcendence, forgiveness, coping, and practices. It is a key domain of resilience recommended in the palliative care of heart failure patients^53^ and with a positive effect on decreasing psychological distress in ICD patients.^54^ This highlights the importance of exploring the extent of integrated care, including access to faith-based support and the recognition of faith as a crucial element in understanding health and well-being.

#### Pharmacotherapy

The pharmacotherapy management of depressive disorders comorbid with cardiac diseases has demonstrated beneficial effects of antidepressants compared to placebo.^55^ Further studies have shown that the use of antidepressants, such as selective serotonin reuptake inhibitors (SSRIs) in combination with psychotherapy, can be effective in reducing the risk of death.^56^ However, antidepressants can affect heart rate, blood pressure, and QT interval, making it essential to carefully review the choice of medication. For instance, tricyclic antidepressants, which have actions like Class IA antiarrhythmics, should be avoided in cases of ischemic origin of SCA due to the risk of ventricular arrhythmias.^57^ Similarly, medications can be useful in managing post-SCA anxiety. SSRIs are effective in treating cardiac anxiety resulting from the dysfunctional interpretation of heart sensations post-ICD-S or SCA. They can also serve as mood stabilizers in these scenarios enhanced by their relatively safe cardiac profile.^58^ Propranolol shows some promise for PTSD prevention^59^ and could be ideal post-SCA and ICD-S due to its concomitant role in acutely managing electrical storms,^60^ although patients' concerns like drug dependency and preferences for nonpharmacological approaches of anxiety management in should be addressed during follow-up. Some rodent studies have shown its potential role in disrupting fear memory reconsolidation in the amygdala, which is a novel method of treating PTSD.^59^ Angiotensin-converting enzyme inhibitors are also associated with decreased depression onset in hypertension patients and are widely used in cardiac patients who might develop SCA.^61^

Drug-drug interactions need to be checked and accounted for, such as the one between fluoxetine and warfarin (CYP2C9 inhibition) and between SSRI/serotonin-norepinephrine reuptake inhibitors and beta-blockers (inhibition of CYP2D6 with decreased beta-blocker activity).^57^ Amiodarone, a CYP3A4 inhibitor, can increase the levels of benzodiazepines such as alprazolam.^57^ Metabolism of flecainide and amiodarone can be inhibited by certain SSRIs (eg, fluoxetine and paroxetine) through inactivation of CYP2D6 and CYP3A4, respectively, leading to toxicity.^57^ Symptoms to monitor when on combined therapy include sedation, blood dyscrasia, and anticholinergic effects. SSRIs, tricyclic antidepressants, and certain antiarrhythmics like disopyramide have anticholinergic effects, which can lead to increased risk of dry mouth, constipation, and urinary retention. Finally, the neuropsychiatric effects of cardiovascular medications should also be considered. Beta-blockers, digoxin, calcium-channel blockers, and amiodarone have been associated with fatigue and mood disturbances.

## Emerging issues and future directions

Given these varied experiences of and considerations for the MH issues post-ICD-S and SCA, a whole-person approach is necessary. As evidence is increasingly built into standardized treatment pathways, integrated care for psychological assessment and interventions, and inclusion of holistic wellbeing interventions are value-driven visions of SSCA care that can be achieved through a health care systems approach that deliberately incorporates MH and linkages with community partners. Identified gaps at different levels are next discussed and further highlighted in Table 1.

**Table 1:** Identifiable Gaps and Improvement Strategies

Gaps	Strategies to Improve MH Post-SCA or ICD-S
Basic science research	Assess the effect of SCA and ICD-S on neuropsychiatry
Clinical research	Optimal delivery method of MHC. Standardized or validated surveys of survivors and their families to define their perception of gaps and appropriately quantify disease burden perception of gaps and their experience. Optimal time for care initiation timing and length of therapy. Effective modalities (cognitive, psychological, including post-traumatic growth approach, peer groups, spiritual care).
Clinical applications	Determination of the most sensitive psychological and cognitive tools to assess MH. Development of an AI prognostic tool.
Health advocacy and policy	Providing direct services to patient and families after ICD-S or post-SCA. Defining barriers within SDOH Policy support for public health projects
Interprofessional workforce and cotraining models	Training of medical staff on MHC Establish joint committees with expertise in critical care, cardiology and neuropsychiatry in medical societies, hospital and outpatient clinics. Increasing transparency by allowing access to MH records by all specialties without increasing stigma
Alternative payment models	Assess financial determinants of behavioral inequity. Value-based health care with metrics of achieving psychological well-being.
Raising awareness and destigmatizing MH	MH awareness campaigns. Informing patients about helpline resources prior to discharge Educating patients and family regarding MH disorders to avoid bias toward treatment and encourage help-seeking.

Table outlining key gaps in MH post-SCA or ICD-S and strategies aimed at improving access and integration across the health system.

AI = artificial intelligence; ICD-S = implantable cardiac defibrillator-shock; MH = mental health; MHC = mental health care; SCA = sudden cardiac arrest; SDOH = social determinants of health.

Social disparities in MHC can significantly impact patients' recovery and QoL. Thus, routine social health determinants screening during care for SSCA should be performed. Survivors often face psychological challenges but access to MH services varies widely based on socioeconomic status, race, ethnicity, and geographic location. According to the National Institute of Mental Health, patients of minority and lower-income background often encounter greater barriers to receiving appropriate MHC in general, due to factors like stigma, inadequate insurance coverage, and limited provider availability.^62^ Improving equitable access to tailored MHC is critical to enhancing patient outcomes and addressing these systemic disparities. Furthermore, the barriers of sufficient workforce to address the psychological evaluation and care of cardiac patients was recently highlighted and the need for cotraining programs in both cardiology and psychology would likely provide the most benefit.^4^ At the national level, the establishment of cardiac psychology or cardiopsychology as a distinct clinical practice and research field has been recommended^63^ but has still great potential to grow into widespread adoption. However, efforts toward this practice do exist, such as the formation of the Cardiopsychology Work Group by the American College of Cardiology and sparse models of cardiac psychology training.^5^ The initial screening by experts (who can be psychology clinicians trained in cardiology, or cardiovascular clinicians trained in psychology) could confer the ability to identify at-risk survivors early on prior to discharge and initiate a multidisciplinary care inpatient and outpatient. One such “Copenhagen Framework” model developed a robust plan to identify patient's in need as well as providing multidisciplinary approach with occupational therapy, neuropsychology, and cardiology to provide safe transitions of care from the inpatient to outpatient setting.^64^ This model uses early detection of both cognitive and psychological issues to tailor individual needs on the outpatient setting. This emphasizes the need of the field of cardio-psychology, especially in this highly vulnerable group.

Addressing SSCA needs will also effectively require integrating MHC into the post-SCA care pathway nationwide, preferably built on existing infrastructure. This integration would involve regular psychological assessments that could be incorporated into national registries and tailored interventions based on individual needs. Establishing evidence-based practices in this area is crucial.

A recovery-oriented care for inpatient MHC could be the best suitable model for managing these patients. It will allow a safe transition from inpatient to outpatient care, relying on integrated medical care that focuses on patient's well-being and propelling his social inclusion and community participation. This will mean adjusting care for each survivor's personal needs and social situation and focusing on community support as well as regular evaluation. In the presence of a suitably built electronic health record, the necessary data can be captured and used by billers to request payment for this model. A value-based compensation model could be proposed. It is contemplated that such a model would rely on assessment and documentation of patient's improvement in physical, mental, and social well-being in subsequent visits.

**Figure 2:**
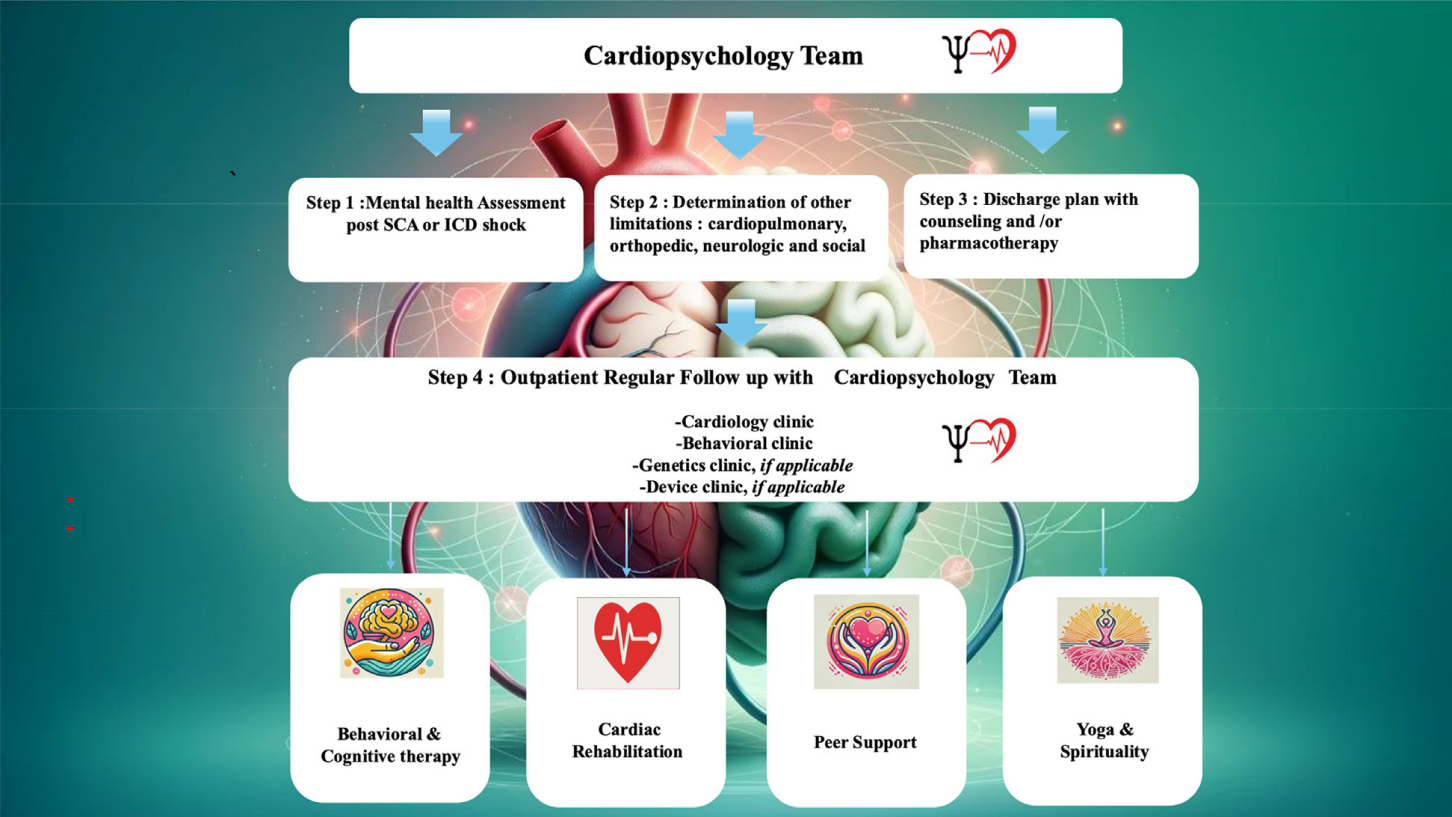
Inpatient and Outpatient Mental Health Care in Survivors of Sudden Cardiac Arrest and Post-ICD-S: A Practical Guidance Figure illustrating inpatient and outpatient mental health care for SCA and ICD shock survivors, featuring cardiopsychology-led assessment prior to discharge and on follow-up peer and spiritual support, behavioral therapy, and rehabilitation. ICD = implantable cardiac defibrillator; SCA = sudden cardiac arrest.

Finally, professional organizations and policymakers could serve a crucial role in establishing and promoting such programs so that a scalable system for psychological well-being can be implemented at the population level (Figure 2) and improving the population physical health.

## Conclusions

SCA and ICD-S can affect the MH of survivors, which may increase mortality and reduce QoL. It is crucial to reduce the burden of MH associated with these events to enhance care delivery and outcomes for this high-risk population by training health professionals, supporting multidisciplinary efforts, and implementing strategies to identify and manage MH sequelae. These efforts when guided by a recovery-oriented care could impact the overall well-being of the patient and be a major step into establishing a health care system with a comprehensive vision of healing in a value-based care context.

## Funding support and author disclosures

Dr Sears has served as a research consultant on quality of life to Medtronic, Abbott, Thryve, Solid Biosciences, Philips, and Milestone Pharmaceuticals; has received a research grant from CVRx that is paid to East Carolina University; and has received speaker honorarium from Medtronic and Zoll Medical in the last year. Dr Mena-Hurtado has served as a consultant for Terumo and has received research grants from 10.13039/100004334Merck and Shockwave. Dr Smolderen has served as a consultant for Terumo, Happify, and Novo Nordisk; and has received research grants from 10.13039/100004334Merck, 10.13039/100004331J&J, Shockwave, and 10.13039/100000046Abbott. All other authors have reported that they have no relationships relevant to the contents of this paper to disclose.
